# Reinforcement Learning–Based Digital Therapeutic Intervention for Postprostatectomy Incontinence: Development and Pilot Feasibility Study

**DOI:** 10.2196/83375

**Published:** 2026-02-06

**Authors:** Fan Fan, Hao Huang, Jingwen Yan, Chao-Yue Xu, Xiuhua Wu, Chunmei Zhou, Dandan Wen, Hai Huang, Ho Cheung Li, Yihong Qiu

**Affiliations:** 1 Department of Urology Sun Yat-sen Memorial Hospital Sun Yat-sen University Guangzhou, Guangdong China; 2 The Nethersole School of Nursing Faculty of Medicine Chinese University of Hong Kong Hong Kong China (Hong Kong); 3 Department of Nursing Sun Yat-sen Memorial Hospital Sun Yat-sen University Guangzhou, Guangdong China

**Keywords:** behavioral rehabilitation, clinical decision support, digital therapeutics, mobile health, pelvic floor muscle training, prostate cancer, quality of life, reinforcement learning, self-management, telemedicine, urinary incontinence

## Abstract

**Background:**

Postprostatectomy incontinence (PPI) is a common complication after robot-assisted radical prostatectomy and significantly impairs patients’ quality of life. Although behavioral interventions such as pelvic floor muscle training and bladder diaries are evidence-based, their effectiveness is often limited by poor adherence and lack of personalization.

**Objective:**

This study aimed to develop and evaluate a reinforcement learning (RL)–driven clinical behavioral intervention-supporting system (CBISs) for adaptive, personalized rehabilitation in patients with PPI.

**Methods:**

The study comprised 2 sequential stages. First, the CBISs was developed through (1) construction of a medical record database from a prospective cohort of PPI patients using standardized 3-day bladder diaries, (2) design of functional modules and user interfaces based on clinical rehabilitation needs, and (3) development of an RL model using XGBoost (extreme gradient boosting) and Bayesian optimization to generate individualized training plans. Second, a separate cohort of 16 patients participated in a single-arm, pre-post pilot study to evaluate feasibility and preliminary outcome trends over a 3-month intervention period, with assessments based on bladder diary parameters and system usage metrics.

**Results:**

The CBISs successfully implemented an adaptive, closed-loop behavioral rehabilitation framework that dynamically tailored training recommendations according to individual voiding patterns, fluid intake behaviors, and adherence signals. Feasibility outcomes were favorable, with high system engagement observed throughout the intervention (mean usage frequency 5.2, SD 1.1 times per day). In exploratory pre-post analyses (n=16), consistent directional improvements were observed across multiple outcomes. Mean daytime urinary frequency decreased from 5.74 (SD 1.21) episodes per day to 4.69 (SD 1.08) episodes per day, while median nighttime urinary frequency declined from 1.8 (IQR 1.6-2.2) episodes per night to 1.0 (IQR 1.0-1.6) episodes per night. Median incontinence episodes were reduced from 7.0 (IQR 6.0-11.0) episodes per day to 4.0 (IQR 2.0-6.0) episodes per day. Objective urine leakage measured by the 1-hour pad test decreased from a median of 8.5 (IQR 4.0-19.0) g to 3.5 (IQR 2.0-9.0) g. Patient-reported symptom burden, assessed using the International Consultation on Incontinence Questionnaire–Short Form (ICIQ-UI SF), showed a median reduction from 14.0 (IQR 12.0-20.0) points to 9.0 (IQR 6.0-16.0) points. Although several within-participant changes were statistically detectable, effect magnitudes varied across individuals. Given the single-arm design, small sample size, and lack of a control group, findings are presented as exploratory and hypothesis-generating rather than confirmatory of clinical efficacy.

**Conclusions:**

The CBISs represents the first RL-powered digital therapeutic system for PPI, enabling adaptive, evidence-based behavioral optimization. By addressing limitations of static rehabilitation protocols and declining adherence, it offers a scalable approach for personalized PPI management. Future multicenter trials are needed to confirm its clinical effectiveness.

## Introduction

Prostate cancer (PCa) is one of the most prevalent malignant tumors in men and currently represents the second leading cause of cancer-related mortality worldwide [1]. It is estimated that by 2025, approximately 313,780 new cases of PCa will be diagnosed in the United States, accounting for 15.4% of all newly diagnosed cancers [2]. Over the past decade, robot-assisted radical prostatectomy has been increasingly recognized as a primary treatment for localized PCa [3]. Owing to the removal of the prostate, seminal vesicles, and surrounding tissues, as well as the reconstruction of the urinary tract during surgery, patients frequently experience varying degrees of postoperative bladder dysfunction. Among these complications, postprostatectomy incontinence (PPI) is the most common functional sequela, severely affecting patients’ daily activities, sexual function, and overall quality of life (QoL) [4-6]. The reported incidence of PPI varies widely, ranging from approximately 4.2% to 87%, largely due to differences in definitions of urinary incontinence, follow-up duration, and assessment methodologies [7]. PPI symptoms typically persist for 3-12 months and, in some cases, may extend for up to 10 years, rendering urinary incontinence a major long-term concern during postoperative recovery [8,9]. Consequently, improving PPI and related QoL outcomes remains a central objective in the comprehensive management of survivors of PCa [10].

Behavioral therapies, including pelvic floor muscle training (PFMT), bladder retraining, urge suppression techniques, and lifestyle modification, constitute the cornerstone of conservative management for urinary incontinence. High-quality evidence from systematic reviews and meta-analyses, including Cochrane reviews, has consistently demonstrated that behavioral therapy–based interventions can reduce incontinence episodes and improve patient-reported QoL across diverse populations [11,12]. These interventions are widely recommended as first-line or adjunctive strategies in clinical practice, reflecting their favorable safety profiles and broad applicability. Nevertheless, the effectiveness of behavioral rehabilitation in real-world settings is frequently compromised by inadequate adherence, limited access to professional supervision, and insufficient patient understanding of correct training techniques.

Advancing from this foundation, digital therapeutics (DTx) have emerged as a promising modality for delivering structured rehabilitation programs remotely, thereby improving accessibility and continuity of care for patients following postprostatectomy. DTx are interventions delivered via digital platforms [13], which have the potential to mitigate the challenges posed by chronic diseases, such as enhancing patient adherence and improving patient self-management [14]. By enabling real-time monitoring, personalized feedback, and flexible scheduling, digital platforms may help address many of the practical limitations inherent in traditional rehabilitation models [15]. However, many existing digital interventions for urinary incontinence remain largely static, relying on predefined rules or uniform training protocols that do not adequately account for individual variability in symptoms, adherence patterns, or recovery trajectories.

In this context, reinforcement learning (RL) represents a promising methodological framework for advancing personalization within digital therapeutic systems. RL is a machine learning approach whereby intelligent agents iteratively optimize decision-making models through cycles of action, feedback, and strategy adjustment [16]. Unlike traditional supervised learning methods, RL is particularly well suited to sequential decision-making problems where the effects of an action may be delayed, outcomes are uncertain, and the optimal strategy depends on the evolving state of the individual. In digital health applications, RL has been used to personalize “just-in-time” interventions—for example, adapting the timing, content, or intensity of behavioral prompts—based on each patient’s history, context, and response pattern, thereby improving engagement and clinical outcomes compared with static or rule-based approaches [17]. Moreover, RL models are capable of processing sparse or noisy reward signals arising from heterogeneous patient behaviors and complex intervention-response relationships, making them especially suitable for individualized rehabilitation scenarios [18]. Applying it to DTx may facilitate individualized patient rehabilitation training.

In this study, to address the need for personalized rehabilitation, a Clinical Behavioral Intervention-Supporting System (CBISs) was developed. Using RL, the CBISs provides adaptive, individualized training guidance to patients with urinary incontinence following robot-assisted radical prostatectomy, aiming to accelerate functional recovery and improve QoL through data-driven support.

Therefore, the objectives of this study were to develop and preliminarily evaluate an RL–based CBISs integrated into a digital therapeutic platform to provide individualized pelvic floor and behavioral training for men with PPI after robot‑assisted radical prostatectomy. The study was designed to examine whether an RL-driven CBISs could (1) demonstrate feasibility and acceptability, with favorable trends in urinary continence recovery and patient-reported QoL; and (2) enhance adherence to and engagement with the prescribed training over time.

## Methods

### Overview

The study was structured in two sequential phases, (1) the development of the CBISs, and (2) a feasibility and preliminary efficacy pilot test of the CBISs conducted with a separate cohort of 16 patients. The development of the CBISs consisted of three main stages, (1) construction of a clinical behavioral database, (2) design of system functionalities and user interfaces based on rehabilitation needs, and (3) development and integration of RL models to support individualized training.

### Recruitment of Participants

All patients were recruited during the follow-up visit of the Outpatient Department of Urology at Sun Yat-sen Memorial Hospital, Sun Yat-sen University. Patients were eligible if they were aged 18-75 years, had a confirmed diagnosis of urinary incontinence following radical prostatectomy, and could accurately maintain a voiding diary (recording fluid intake, urination, and leakage). Exclusion criteria comprised serious neurological diseases, concurrent conditions such as urinary tract infection or bladder stones, significant voiding dysfunction, and an inability to comply with the study protocol. During the CBISs construction phase, we recruited a total of 150 patients to participate.

### Database Construction

This section details the complete workflow for building the analytical database, including the statistical procedures and tests for data curation and feature engineering, which collectively form the essential foundation for the subsequent development of the prediction model. Bladder diaries were prospectively collected from patients diagnosed with PPI at the urology department of a tertiary care academic hospital. To ensure data robustness, a minimum diary period of 3 days was required based on International Continence Society standards for reliable symptom capture [19].

Sample size determination addressed two dimensions. At the patient level, sample size considerations focused on ensuring sufficient coverage of behavioral variability for model development rather than hypothesis testing. A cohort of 150 patients was deemed adequate to support feature engineering, model training, and internal validation while minimizing the risk of overfitting. At the behavioral event level, more than 10,000 voiding events were targeted to ensure model generalizability, accounting for expected intrapatient variability (mean, 8 voids per day per patient). Sample size considerations therefore accounted not only for the number of patients but also for the volume of behavioral data, with a 3-day behavioral log per patient ensuring minimum model generalizability. Ethical approval for the collection of data required for system construction was obtained from the Medical Ethics Committee of Sun Yat-sen Memorial Hospital, Sun Yat-sen University (approval number: SYSKY -2023-925-01). Data collection was conducted between March 2022 and December 2023.

### Determination of Key Features and System Functionalities

The research team conducted a systematic literature review and held 2 rounds of expert meetings to develop a checklist for the rehabilitation needs of patients with PPI. Based on this, researchers and software engineers collaborated to define the functional module division and design the human–computer interaction interface. The CBISs comprises 3 portals: patient portal, a health care team portal, and an administrator portal. The patient portal contains 4 modules: rehabilitation training, bladder diary, incontinence care, and assessment tool. The health care team portal displays the patient’s bladder diary details and training completion status.

### Reinforcement Learning Model Construction

The research team developed the conceptual plan and proposed the functional requirements of the model; algorithm engineers then constructed and validated the training model, which was subsequently embedded into the CBISs to complete the design and development. Individualized rehabilitation training models for patients with PPI include urination training, fluid intake training, and PFMT. To realize these features, the algorithm engineers used a preconstructed database and performed feature screening and dimensionality reduction through an exhaustive feature engineering step.

### Data Preprocessing and Quality Control

Prior to model training, all raw bladder diary records and behavioral logs underwent a structured preprocessing pipeline. Missing values in continuous variables (eg, voided volume and urination interval) were imputed using patient-level mean strategy, while discrete variables (eg, fluid type and urgency score) adopted mode imputation. Abnormal values were detected through IQR filtering and time-series consistency checks. All timestamps were normalized to standard 24-hour format, and temporal alignment of drinking and voiding events was ensured using sliding-window techniques with a 60-minute threshold. Records from patients with <80% diary completion rate or >20% anomalous entries were excluded from analysis to guarantee robust data quality and integrity.

### Feature Selection and Model Training Strategy

From the initial 29 behavioral and physiological features, recursive feature elimination and XGBoost’s (extreme gradient boosting’s) intrinsic gain-based importance ranking were jointly applied to refine input dimensions. Highly collinear features (Pearson *r*>0.9) were removed to prevent multicollinearity. Model development was performed using a stratified 70/15/15 train/validation/test split, preserving the distribution of leakage severity and adherence levels. Bayesian optimization was used to fine-tune hyperparameters (eg, learning rate, max_depth, and subsample) with the goal of maximizing prediction accuracy and minimizing overfitting. Model performance was evaluated using a composite metric, including area under the receiver operating characteristic curve (AUC), *F*_1_-score, and mean absolute error (MAE). A 5-fold cross-validation strategy was used during the training phase to ensure model generalizability and robustness.

### Reinforcement Learning Framework and Deployment

The customized offline RL architecture is adopted in the CBISs to support adaptive behavioral rehabilitation while clinical safety is maintained. The RL component is designed to function as a decision-support layer rather than as an autonomous optimizer of clinical outcomes, reflecting the exploratory nature of this pilot study.

Clinical state representations are constructed from multiday behavioral snapshots encompassing key metrics such as PFMT adherence, voiding intervals, fluid intake ratios, and patient-reported outcomes. These inputs closely correspond to the information typically evaluated by clinicians when adjusting rehabilitation strategies, thereby ensuring that the RL state definition remains clinically interpretable and grounded in routine practice.

At each decision point *t*, the system observes a behavioral state *s_t_* and selects an action *a_t_* from a predefined discrete action space. Actions correspond to clinically admissible behavioral interventions; for example, when a patient demonstrates stable adherence to the prescribed training program but reports repeated episodes of urinary leakage, the system may recommend a modest extension of PFMT duration (eg, +15 minutes) while maintaining the current training intensity. Subsequent adjustments to PFMT intensity are then guided by changes in the frequency of reported leakage, allowing the intervention to progress in a gradual and clinically responsive manner. All actions are constrained within predefined safety ranges to avoid abrupt or excessive changes.

### Reward Function Formulation

The reward function is designed to reflect clinically meaningful behavioral changes rather than to directly optimize long-term clinical outcomes. At each decision point *t*, the reward *r_t_* is computed as a weighted composite function:



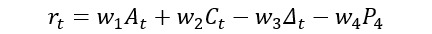



where *A_t_* represents short-term adherence to prescribed training tasks, *C_t_* reflects task completion and user engagement, *Δ_t_* captures the magnitude of abrupt behavioral changes between consecutive recommendations, and *P_t_* denotes penalty terms applied when predefined safety thresholds are approached (eg, overly frequent voiding prompts or rapid escalation of training schedules). All components are normalized to bounded ranges prior to aggregation to ensure numerical stability and interpretability.

In practical clinical terms, this formulation supports gradual and responsive rehabilitation adjustments. For instance, when a patient reports an increase in urinary leakage episodes, the system may recommend extending PFMT duration by 15 minutes, representing a moderate increase in task demand. If the patient demonstrates high adherence and engagement following this adjustment and a subsequent reduction in leakage frequency is observed, penalty terms decrease and the revised training plan is maintained. Conversely, if task completion or engagement declines and leakage frequency fails to improve, penalty values increase, indicating that the adjustment may be poorly tolerated. In such cases, the system may revise the recommendation by reducing the training extension (eg, to an additional 5 minutes) and continue monitoring adherence, engagement, and leakage trends before further modification. This iterative process mirrors routine clinical reasoning, emphasizing tolerability, patient response, and safety rather than rigid optimization.

### Q Value Estimation and Policy Learning

Based on the defined reward, action values are estimated using standard Q-learning principles. The Q value represents the expected cumulative future reward of selecting action *a_t_* ​under state 𝑠_𝑡_ and is updated offline according to:



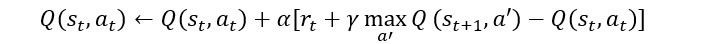



where *α* is the learning rate and *γ* is the discount factor. Q values are learned entirely from historical behavioral data using offline batch updates, without real-time exploration in patients.

Conceptually, Q values estimate how beneficial a specific behavioral adjustment is expected to be over time. Actions associated with higher Q values are more likely to be recommended, as they have historically led to stable adherence and acceptable behavioral patterns.

To handle variability in patient engagement and avoid overreliance on historical patterns, an ε-greedy action selection strategy is used. In most situations, the system selects the action with the highest estimated Q value; however, with a small probability ε, alternative actions are explored. In simple terms, the model usually follows what has worked best before, but occasionally tests other reasonable options to prevent overly rigid behavior, particularly for patients with inconsistent adherence.

### System Usability and Technical Validation

Usability testing was conducted with a pilot group of 30 patients drawn from the database construction cohort, recruited from September to November 2023, who participated in a 4-week trial from December 2023 to January 2024. Task completion rates, interaction time, feedback scores, and daily user feedback during usage were systematically recorded. The mean training plan navigation time was 56 seconds (SD 12 seconds), with a >92% completion rate of daily bladder diaries. The CBISs demonstrated full functional compatibility with both Android (v8.0+) and iOS (v13+) platforms. Backend performance stress testing indicated stable response under 1000 concurrent users with <500 ms latency. The final release version underwent security penetration testing to ensure robustness against common cybersecurity threats, such as injection attacks, data breaches, and unauthorized data access attempts.

### Integration of Human–Artificial Intelligence Shared Decision-Making

To reinforce clinical safety and ensure alignment with real-world care standards, a “human–artificial intelligence (AI) shared decision-making” mechanism is integrated into the CBISs. This module operates as a safety net for low-confidence or guideline-sensitive scenarios, complementing the core RL model.

Specifically, when the model generates a recommendation with low predictive certainty or when the patient presents high behavioral variability or complex comorbidities, the CBISs triggers clinician review. Health care professionals evaluate the suggestion in a semiautomated interface, which also displays relevant confidence scores and patient history snapshots.

In addition, a rule-based expert constraint layer is provided, allowing clinicians to embed patient-specific limitations such as contraindications, comorbidity exclusions, or institutional guidelines (eg, American Urological Association 2024 and International Continence Society). All final behavior plans are generated through an iterative approval flow that synthesizes algorithmic output, patient feedback, and clinician judgment.

This human–AI collaboration mechanism improves accountability, enhances clinical trustworthiness, and ensures DTx remain aligned with evidence-based care pathways.

### Pilot Test of Clinical Behavioral Intervention-Supporting System

This pilot study was designed as an exploratory, single-arm, self-controlled evaluation to assess feasibility, adherence, and outcome trends rather than to establish definitive clinical efficacy. A separate cohort of 16 patients with PPI was recruited, distinct from the 150 participants was enrolled in the database construction phase. Participant recruitment and CBISs implementation occurred between January 2024 to March 2024. Participants eligibility criteria were consistent with the formal research protocol. Patient age and time since surgery were recorded. Voiding characteristics, including timing, intervals, and nocturia episodes (both frequency and total volume) were systematically collected. Patient compliance was evaluated based on completion of prescribed CBISs diary entries, with actual usage frequency tracked as the adherence metric. All participants were enrolled to use only the CBISs for postoperative rehabilitation for 3 months.

Both baseline data collection and postintervention outcome assessment were conducted over 5-day periods, with the mean values of these 5-day measurements used as pre- and postintervention results for comparison. The following parameters were recorded: daily voided volume (mL), daily fluid intake (mL), daytime urinary frequency, nighttime urinary frequency, incontinence episodes, urine leakage measured by the 1-hour pad test (g), total score of the ICIQ-UI SF (range 0-21), daily occurrence of urinary urgency, sensation of incomplete bladder emptying, postvoid dribbling, and dysuria (recorded as present or absent).

For each participant, the mean preintervention value was calculated as the arithmetic average of the baseline observations (the first 5 days of recording), yielding the preintervention daily average. The mean postintervention value was derived similarly from the final 5 days of recording. For symptom data recorded as binary outcomes (present=1, absent=0), symptom frequencies were calculated as continuous variables. Preintervention symptom frequency was defined as (number of days the symptom was present during the 5-day baseline period) / 5 × 100%. Postintervention symptom frequency was defined as (number of days the symptom was present during the final 5-day intervention period) / 5 × 100%. For all measured parameters, the change score (Δ) for each participant was computed as: Δ = postintervention value – preintervention value.

All statistical analyses were conducted using R software (version 4.4.2; R Foundation for Statistical Computing). Given the exploratory nature of this pilot study, analyses were performed to describe within-participant change patterns and estimate effect magnitude rather than to test confirmatory hypotheses. A 2-tailed α level of .05 was adopted for descriptive purposes only.

The distribution of individual change scores (Δ) was assessed using the Shapiro-Wilk test. Paired-sample 2-tailed *t* tests or Wilcoxon signed-rank tests were applied as appropriate based on distributional assumptions. Binary symptom variables were converted to symptom frequency percentages and analyzed as continuous outcomes when normality assumptions were met.

Spearman rank correlation coefficient was used to explore associations between changes in selected outcomes. No adjustment for multiple comparisons was performed due to the pilot design and limited sample size, and statistical findings should be interpreted cautiously.

### Ethical Considerations

Ethical approval for data collection required for system construction was obtained from the Medical Ethics Committee of Sun Yat-sen Memorial Hospital, Sun Yat-sen University (approval number: SYSKY-2023-925-01). All participants provided electronic informed consent approved by the Medical Ethics Committee (SYSKY-2023-925-01), explicitly detailing (1) bladder diary and behavioral data collection for RL model training to generate personalized recommendations, (2) anonymized data use for system-wide model improvement, (3) right to withdraw data or consent at any time without impact on care, (4) potential implications of AI-driven personalization (eg, adaptive vs static guidance). Consent forms used plain language with examples and were available in Chinese and English.

To mitigate bias from incomplete or anomalous data, preprocessing excluded records with <80% diary completion or >20% anomalies (IQR filtering and time-series checks). Stratified sampling (70/15/15 train/validation/test) preserved leakage severity and adherence distributions. Model fairness was monitored through subgroup performance (age and time since surgery) during 5-fold cross-validation, with clinician override for low-confidence predictions ensuring equity.

For data security, uploaded records were protected using ShangMi 3 (SM3; a cryptographic hash algorithm for integrity verification) and ShangMi 4 (SM4; a symmetric block encryption algorithm for secure data storage and transmission), in accordance with national cryptographic standards. Data handling adhered to China’s Personal Information Protection Law and the General Data Protection Regulation–equivalent standards for health data. Health care professionals accessed aggregated views through the CBISs using an authorization key. Additionally, patient-side interfaces integrated real-time alerts for suspicious login attempts or data anomalies, ensuring prompt intervention and maintaining data integrity and patient privacy.

## Results

### Demographic and Disease-Related Characteristics of Participants

In the first phase of this study, a total of 150 participants were included who provided data for system development, model construction, and demographic characterization. From this cohort, a subset of 30 additional participants was selected to participate in the focused usability testing phase of CBISs.

The full modeling cohort (N=150) had an age range of 43-82 years, with a mean age of 54.32 (SD 7.51) years. Participants included in the usability testing subsample (n=30) showed a comparable age distribution, ranging from 43-81 (mean 53.70, SD 7.30) years.

Regarding educational attainment, the modeling cohort was predominantly composed of 46 (30.67%) individuals with junior high school education, followed by 35 (23.33%) participants with vocational school education, 28 (18.67%) individuals with undergraduate education, 22 (14.67%) with senior high school education, 12 (8.00%) with primary school education, 6 (4.00%) with postgraduate education, and 1 (0.67%) with no formal education.

In the usability testing subsample, junior high school education was the most prevalent, reported by 13 (43.33%) participants, followed by junior college education with 6 (20%) participants. Senior high school and primary school education were each represented by 4 (13.33%) participants, while vocational school education accounted for 2 (6.67%) participants, and undergraduate education for 1 (3.33%) participants. No participants reported postgraduate education or no formal schooling.

With respect to physical characteristics, the mean BMI of the modeling cohort was 23.44 (SD 3.11) kg/m^2^. The usability testing subsample demonstrated a similar BMI distribution, with a mean value of 24.01 (SD 3.18) kg/m^2^. Overall, participants included in the usability testing phase were broadly comparable to the full modeling cohort in terms of age and BMI, while exhibiting modest differences in educational composition.

In the pilot test of CBISs, participants used the CBISs an average of 5.2 (SD 1.1) times per day. A total of 11 (68.75%) participants met or exceeded the preset usage target, while 5 (31.25%) participants fell below the target, with a minimum usage of 3 times per day. This indicates generally high engagement with the system during the intervention period. All participants, being older adults, ranged in age from 61-74 years and had a mean age of 67.6 (SD 4.3) years. Among them, 11 (68.75%) participants were within 1-3 months after PCa surgery, and 5 (31.25%) participants were within 3-6 months postoperatively.

### The Model of Clinical Behavioral Intervention-Supporting System: Feature Engineering

Based on the patient bladder diary and behavioral monitoring data, a total of 29 features in 6 categories were constructed in this study (Table 1), with the feature construction methods detailed in Table 2. Following data cleaning, the dataset was partitioned via stratified sampling. Model construction was performed based on XGBoost, incorporating Bayesian optimization for model and parameter tuning.

**Table 1:** Patient data collection form for clinical behavioral intervention-supporting system (CIBSs).

Data category			Instruction
**Basic information**			
	Gender	Discrete variables (male/female)	
	Age	Continuous variable (years)	
	BMI	Continuous variable (kg/m^2^); associated with symptom typing, behavioral competence, and training intensity appropriateness	
	Time of onset	Continuous variable; determines symptom chronicity	
	Urinary flow rate parameter (Q_max_)	Continuous variable; identifies storage, voiding disorders, or bladder hypo-compliance, etc	
	Urinary flow rate parameter (Q_ave_)	Continuous variable	
	Residual urine volume test records	Continuous variable	
**Urinary behavior**			
	Time of urination	Continuous variable (min); establishment of a 24-hour urinary rhythm	
	Urinary output	Continuous variable (mL); partially collected automatically by portable urine flow rate instrumentation	
	Urinary flow rate	Continuous variable (mL/min); automatically collected by portable urinary flow rate instruments	
	Urination interval	Continuous variable (min); imputed from voiding time difference	
	Nocturnal urination	Continuous variable (times/mL); includes nocturnal urine frequency and volume	
	Urinary urgency event	Continuous variable (grade); graded 0-5, subjectively scored by the patient	
	Symptoms associated with urination	Discrete variables, for example, presence of “urine odor,” “slow urine flow,” “painful urination,” “burning sensation,” “divergent urinary stream,” “feeling of not emptying,” “dribbling after urination,” and so on	
**Drinking behavior**			
	Drinking time	Continuous variable (specific time); establish temporal linkage with urination time	
	Drinking volume	Continuous variable (mL)	
	Drinking type	Discrete type variable, for example, water, tea, coffee, functional drinks, etc	
**Daily activity and sleep behavior**			
	Activity intensity (steps and body movement)	Continuous variable (steps/kcal); from wearable device or mobile health app	
	Activity period	Discrete variable (daytime/nighttime);	
	Sleeping time/number of night arousals	Continuous variable (min/times); basis for determining the rhythm between nocturia and nighttime arousal	
**Specialty scales and subjective scoring data**			
	Overactive Bladder Syndrome Score	Continuous variable; quantitative grading of symptoms to identify overactive bladder	
	International Prostate Symptom Score	Continuous variable; determination of symptom severity during storage/voiding phase	
	International Consultative Committee on Incontinence Questionnaire Short Form	Continuous variable; incontinence symptoms and quality of life implications	
	Self-assessment Scale for Anxiety and Depression	Continuous variable; assess anxiety and depressive states	
	Pittsburgh Sleep Quality Index–Sleep Quality Score	Continuous variable; determine the extent to which nocturia affects sleep	
**System interaction and training feedback data**			
	Record of training completion	Whether bladder training, water intake program, relaxation training, pelvic floor muscle training, etc, are completed as planned	
	Patient feedback score	Daily record of perceived efficacy, difficulty, and compliance	
	Abnormal events	Such as symptom exacerbation or incomplete training prompts	
	Frequency of use, number of times of punching clock	Judge adherence and compliance level	

**Table 2:** Feature engineering methodology for clinical behavioral intervention-supporting system (CIBSs).

Feature category and name		Source data items	Construction method	Clinical significance
**Temporal features^a^**				
	Daytime mean voiding interval	Urination time, day/night encoding	Mean time difference between voids during 9 AM to 9 PM	Evaluates bladder storage stability
	Nocturnal polyuria ratio	Nocturnal urine volume, 24-hour total volume	∑ (nocturnal urine volume) / ∑ (24-hour urine volume) × 100%	Core diagnostic indicator for nocturnal polyuria
	Urgency event frequency	Urgency event markers	Voids with urgency (7 days) / total voids (7 days)	Quantifies overactive bladder severity
**Ratio features^b^**				
	Nocturia-arousal association index	Nocturnal voids, sleep interruptions	(Voided volume) / (voided volume + postvoid residual) × 100%	Differentiates pure nocturia from sleep disorders
	Effective voiding rate	Voided volume, postvoid residual	Nocturia episodes / sleep interruptions	Reflects bladder emptying efficiency
	Irritant beverage intake ratio	Fluid type, intake volume	Coffee + tea intake (mL) / total intake (mL) × 100%	Assesses bladder irritant exposure
**Time-period features^c^**				
	Workday-weekend voiding pattern difference	Voiding time, date type	Mean weekend voiding interval − mean workday voiding interval	Detects stress-related voiding behavior changes
	Postexercise urgency marker	Activity period, urgency markers	Binary indicator: urgency within 30 minutes after vigorous exercise	Identifies exercise-induced urgency
**Contextual features^d^**				
	Fluid-void temporal association index	Intake time, voiding time	Voids within 60 minutes post intake / total voids	Evaluates bladder hypersensitivity
	PFMT^e^ adherence decay rate	Exercise logs, time	Adherence rate (last 3 days) / adherence rate (week 1)	Quantifies decline in behavioral intervention compliance

^a^Based on mean, extreme, and periodic variations within a fixed time window.

^b^Used for reflecting structural relationships.

^c^Used for behavioral pattern recognition.

^d^Describes behavioral markers triggered by specific conditions.

^e^PFMT: pelvic floor muscle training.

### Model Training and Validation

**Figure 1:**
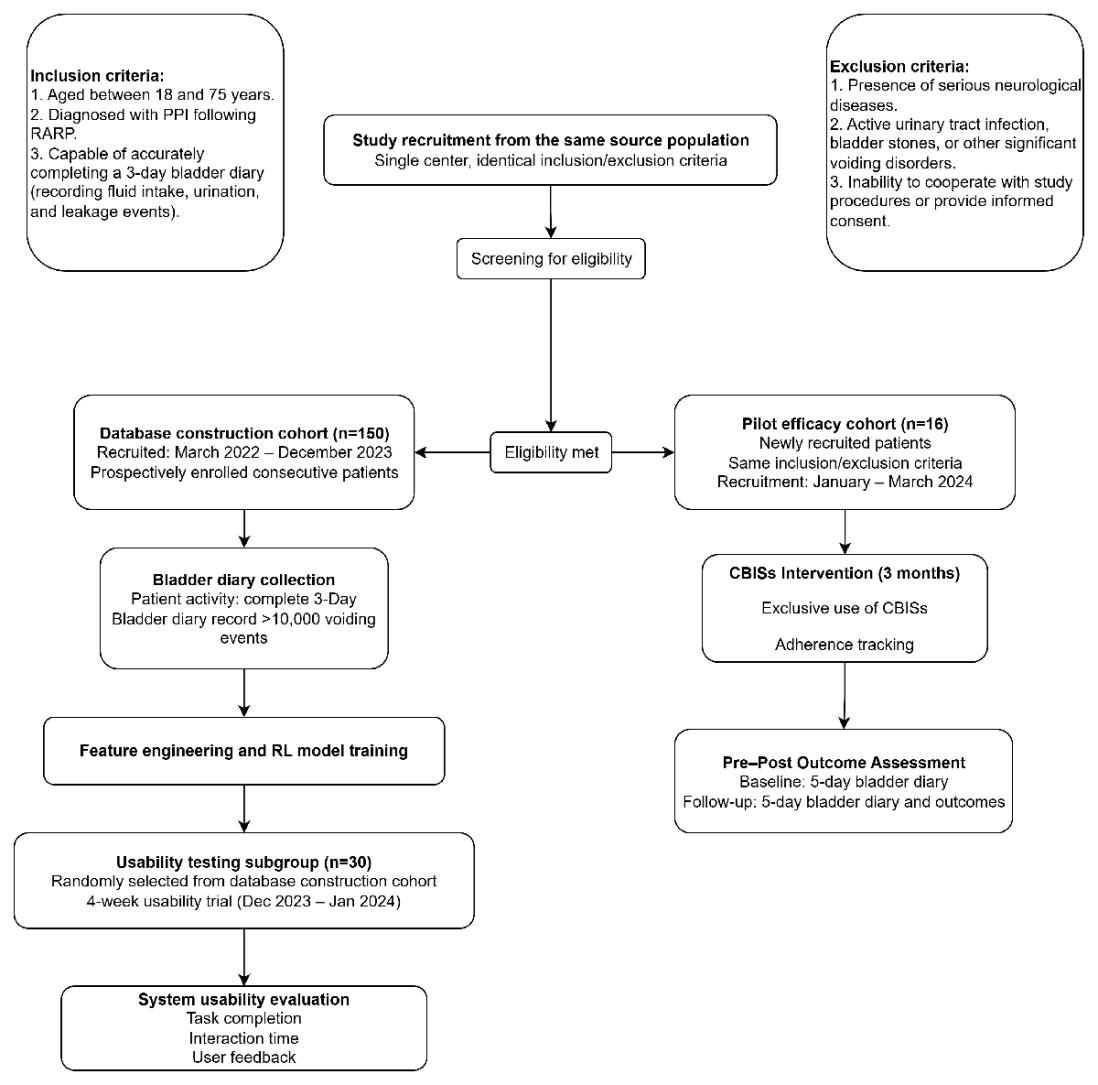
Patient flow diagram for clinical behavioral intervention-supporting system (CBISs) development and pilot feasibility evaluation. PPI: postprostatectomy incontinence; RARP: robot-assisted radical prostatectomy.

The preconstructed database was used to support feature matching and behavioral pattern recognition, which informed the generation of personalized training recommendations. The RL component subsequently updated these recommendations over time based on patient interaction and behavioral feedback, completing the adaptive model construction process (Figure 1).

In the current implementation, the RL framework did not incorporate explicit clinical outcome measures (eg, reduction in leakage episodes or symptom score improvement) as direct reward signals. Instead, reward signals were defined using conservative, behavior-centered proxy indicators, including task completion, adherence stability over time, and avoidance of abrupt behavioral changes potentially associated with symptom aggravation. This design choice was intentionally adopted to ensure clinical safety and model stability in this exploratory pilot setting, where outcome responses are delayed, heterogeneous, and not suitable for short-horizon optimization.

Clinical outcome measures were therefore reserved exclusively for post hoc exploratory evaluation rather than real-time reward optimization. Model evaluation metrics (eg, AUC, *F*_1_-score, and MAE) were used internally during development and tuning to support system calibration but were not reported as final performance outcomes, as this study was not designed as a standalone predictive model validation.

Rather than optimizing a single mathematically fixed reward function, the RL component operated within a clinician-supervised decision-support framework, integrating multiple behavior-related feedback signals under predefined safety constraints. Accordingly, the RL module was designed to support adaptive behavioral guidance within a clinically supervised system rather than to function as an autonomous predictive model intended for direct performance benchmarking.

### The Clinical Behavioral Intervention-Supporting System Mobile App

#### Overview

Based on the literature review and research team meetings, the main functional modules of the CBISs were identified (Figure 1). The CBISs provide patients with personalized voiding schedules, dietary and fluid intake plans, and pelvic floor muscle contraction training to enhance bladder function. A distinctive feature of the CBISs is its consideration of skin-related symptoms potentially caused by incontinence, offering patients guidance on incontinence-related nursing interventions, that is, the incontinence skin care function. Based on their evaluation of perineal skin condition and severity, the CBISs recommends tasks such as daily cleansing, application of skin-protective dressings, and use of incontinence products. The health care team and administrator portals also receive early warning notifications regarding the occurrence of incontinence dermatitis in patients.

#### Key Features and Capabilities

**Figure 2:**
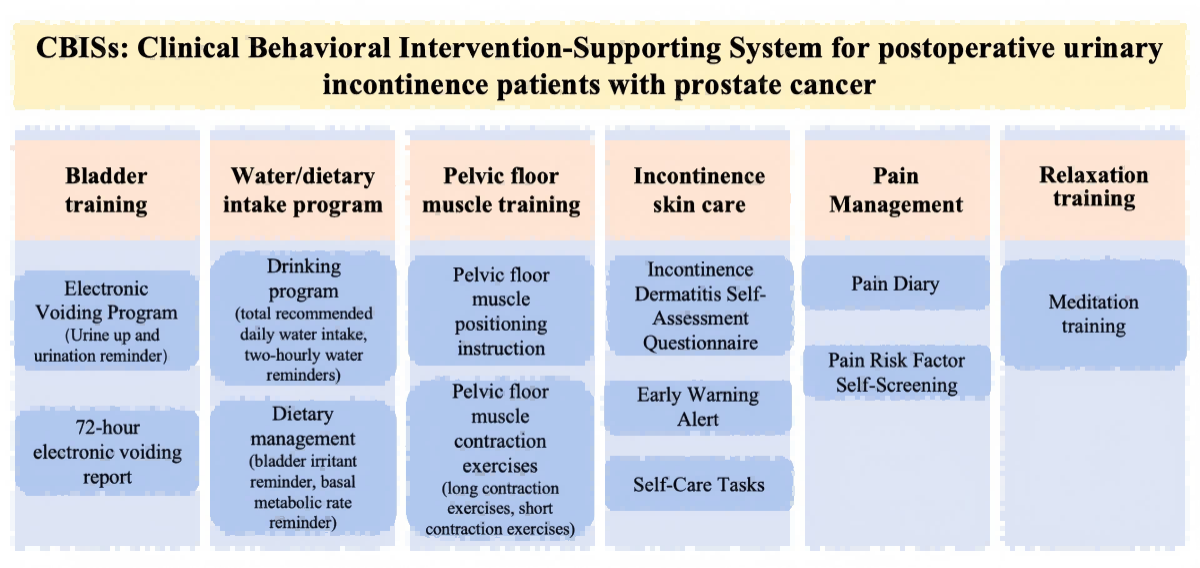
Key functional modules of clinical behavioral intervention-supporting system (CBISs).

The functional sections of the app and its user interface were identified. The app has 2 interfaces: the home page and the personal center. The home page is divided into 4 sections: “Training Sections,” “Bladder Diary,” “Assessment Tools,” and “Incontinence Care.” The “Personal Center” page provides the user’s basic information, contact options for doctors or nurses, and counseling services (Figure 2). Users are required to register an account upon first login and can access the app after using their account number and password. Patients can access the training program after registering their behavioral logs for 3 days.

**Figure 3:**
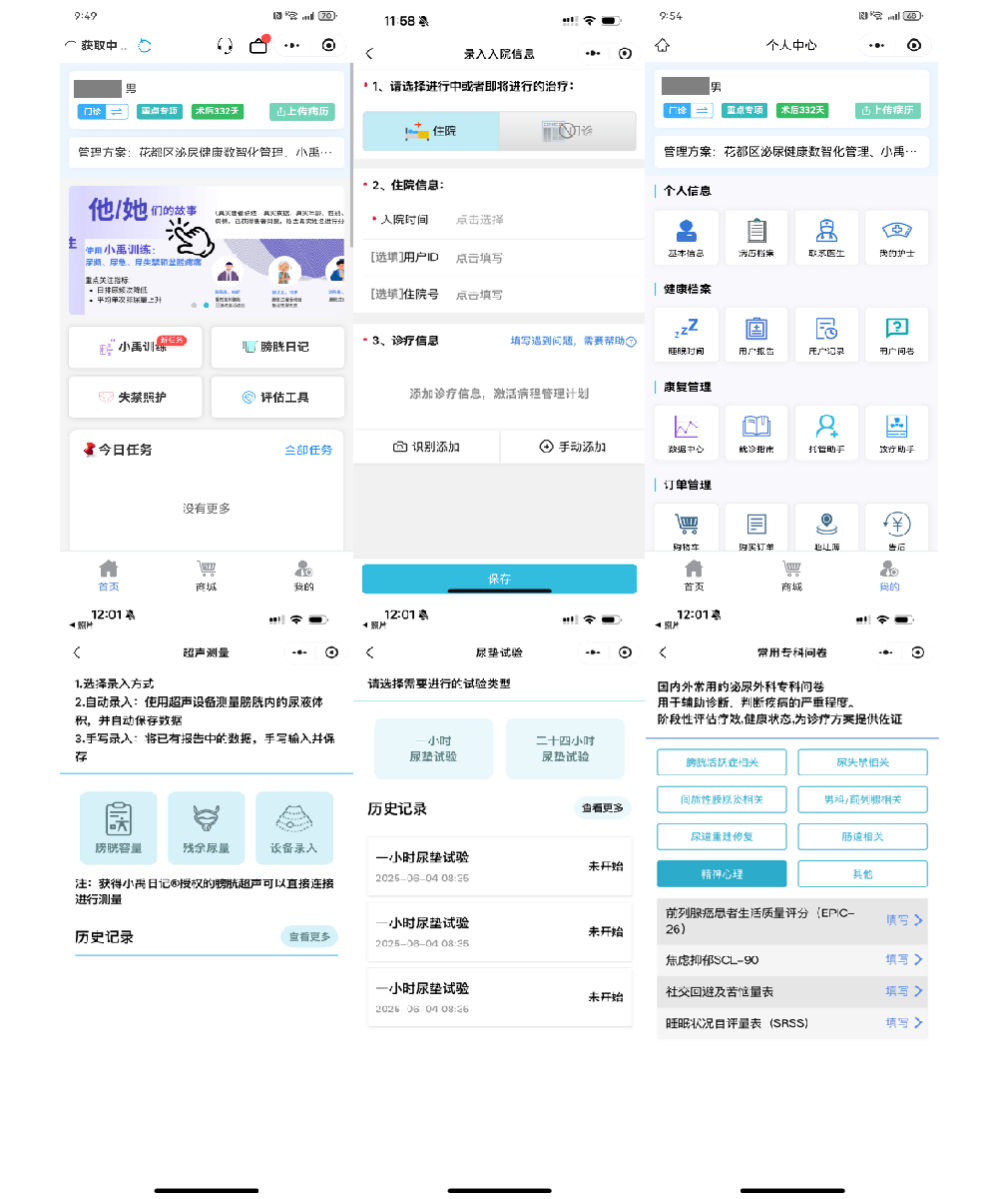
Clinical behavioral intervention-supporting system (CBISs) user interface. (A) Initial app screen—outpatient; (B) initial app screen—inpatient (patient clinical and demographic characteristics); (C) personal homepage (top to bottom: patient overview—Illness severity, demographics, disease details, and responsible medical personnel; records and support—health and user records, alongside user assistance; rehabilitation log—tracks rehabilitation activities and outcomes; service purchase—handles the acquisition of additional services); (D) bladder ultrasound monitoring; (E) urinary pad test; and (F) questionnaire entry screen.

In the training section, the app provides training programs based on the aforementioned RL models. The initial training program is based on the raw data provided by the patient, and the training content specifically includes voiding interval time, drinking time schedules, rising urine training (bladder capacity training), and pelvic floor muscle contraction and relaxation training. Patient’s data are regularly evaluated to assess rehabilitation progress and adjust the training program accordingly. The learning model iteratively adjusts the program, while the health care team may also adjust the program manually. The “Contact Doctor/Nurse” module offers patients support and training guidance throughout the course of their illness to ensure that they maintain good habits after discharge (Figure 3).

#### Safety and Feasibility

Regarding digital confidentiality, patients upload data by completing their own records as well as using wearable devices. Because the data are time series in nature, the app assigns timestamps to each data point and incorporates preprocessing interfaces to filter missing values and outliers. The study follows SM3 and SM4 encryption algorithms for desensitized and encrypted storage of data, alongside hierarchical management of data permissions, facilitating separate review processes for health care professionals and patients. Data privacy complies with the Personal Information Protection Act.

### Pilot Evaluation of Clinical Behavioral Intervention-Supporting System Feasibility and Outcome Trends

Participants used the CBISs an average of 5.2 (SD 1.1) times per day. Following implementation of the CBISs-guided bladder function training program, directional changes were observed across multiple bladder diary parameters, incontinence-related outcomes, and patient-reported symptom measures (Tables 3 and 4). Given the exploratory nature of this pilot study and the limited sample size, results are presented to describe change patterns rather than to support confirmatory inference.

Mean voided volume increased from 984.6 (SD 132.7) mL at baseline to 1128.4 (SD 148.2) mL post intervention. Fluid intake also increased over the intervention period, suggesting changes in hydration and voiding behaviors consistent with the individualized training guidance delivered by the system.

Daytime urinary frequency showed a reduction from 5.74 (SD 1.21) episodes per day to 4.69 (SD 1.08) episodes per day. Nighttime urinary frequency similarly declined, with median values decreasing from 1.8 (IQR 1.6-2.2) episodes per night to 1.0 (IQR 1.0-1.6) episodes per night. Total urinary frequency demonstrated a comparable downward trend over the intervention period, reflecting changes in urinary storage and voiding patterns.

Incontinence episodes declined from a median of 7.0 (IQR 6.0-11.0) episodes per day at baseline to 4.0 (IQR 2.0-6.0) episodes per day after the intervention. Pad test measurements indicated lower postintervention urine leakage values compared with baseline, with a shift toward milder leakage severity categories observed among participants. Specifically, the number of patients classified as having severe or greater leakage decreased, while the number of patients classified as having mild leakage increased.

Patient-reported outcomes showed parallel trends. ICIQ-UI SF scores were lower after the intervention compared with baseline, indicating a reduction in perceived symptom burden. Exploratory correlation analysis suggested alignment between changes in patient-reported symptom scores and reductions in objective urine leakage measures; however, these associations should be interpreted cautiously given the small sample size and exploratory design.

The proportion of patients reporting urinary urgency and sensation of incomplete bladder emptying was lower following the intervention period. Dysuria incidence also declined, whereas changes in postvoid dribbling were less pronounced and more variable across individuals.

Effect size estimates were calculated to describe the magnitude of observed changes across outcomes. Several parameters demonstrated moderate to large effect size values; however, these estimates are provided for descriptive and hypothesis-generating purposes only and should not be interpreted as evidence of definitive clinical benefit in the absence of a concurrent control group.

Overall, the pilot results indicate that the CBISs can be feasibly implemented in postprostatectomy patients and is associated with consistent directional changes across multiple objective and subjective outcome domains. No causal conclusions regarding clinical efficacy can be drawn from this exploratory analysis.

**Table 3:** Exploratory changes in bladder diary parameters before and after the clinical behavioral intervention-supporting system (CBISs)–guided training program. Statistical comparisons are exploratory and intended to describe within-participant change patterns rather than to support confirmatory inference. Effect sizes are reported for descriptive purposes only.

Variable	Preintervention, mean (SD)	Postintervention, mean (SD)	Mean difference, 95% CI	2-tailed *t* test (*df*)	*P* value	Cohen *d*
Voided volume (mL)	984.6 (132.7)	1128.4 (148.2)	143.8 (69.2 to 218.4)	4.32 (15)	<.001	1.08
Fluid intake (mL)	1246.8 (181.4)	1428.2 (198.6)	181.4 (95.6 to 267.2)	5.12 (15)	<.001	1.28
Daytime urinary frequency	5.74 (1.21)	4.69 (1.08)	–1.05 (–1.68 to –0.42)	–3.56 (15)	.003	0.89
Total urinary frequency	7.86 (1.90)	5.96 (1.24)	–1.90 (–2.56 to –1.24)	–6.12 (15)	<.001	1.53
Urinary urgency frequency, %	68.8 (18.2)	31.3 (16.8)	–37.5 (–47.5 to –27.5)	–8.23 (15)	<.001	1.08
Incomplete emptying sensation frequency, %	58.8 (19.5)	28.8 (15.6)	–30.0 (–40.6 to –19.4)	–6.15 (15)	<.001	1.28
Postvoid dribbling frequency, %	25.0 (15.8)	18.8 (12.4)	–6.2 (–14.8 to 2.4)	–1.56 (15)	0.139	0.89

**Table 4:** Exploratory changes in incontinence-related outcomes and patient-reported symptoms before and after the clinical behavioral intervention-supporting system (CBISs)–guided training program. Statistical analyses were conducted to characterize the direction and magnitude of changes in this pilot study. *P* values and effect sizes should be interpreted cautiously due to the small sample size and lack of a control group. For dysuria, the value represents percentage change.

Variable	Preintervention, median (IQR)	Postintervention, median (IQR)	Median difference	*z* value	*P* value	Effect size^a^, *r*
Nighttime urinary frequency, n	1.8 (1.6-2.2)	1.0 (1.0-1.6)	–0.8	–3.82	<.001	0.68
Incontinence episodes, n	7.0 (6.0-11.0)	4.0 (2.0-6.0)	–3.0	–5.56	<.001	0.99
Urine leakage, g	8.5 (4.0-19.0)	3.5 (2.0-9.0)	–5.0	–5.56	<.001	0.99
ICIQ-UI SF^b^ score	14.0 (12.0-20.0)	9.0 (6.0-16.0)	–5.0	–5.32	<.001	0.95
Dysuria incidence, %	43.8	25.0	–18.8*	–3.41	.001	0.61

^a^Effect size interpretation: Cohen *d*: small (0.2), medium (0.5), large (0.8); r effect size: small (0.1), medium (0.3), large (0.5).

^b^ICIQ-UI SF: International Consultation on Incontinence Questionnaire–Short Form.

## Discussion

### Overview

The pilot study provided preliminary evidence of improvements in urinary control and patient-reported symptoms, with the CBISs simulating professional supervision by dynamically adjusting behavioral prescriptions based on adherence patterns, symptom trajectories, and real-time data. Pilot results indicated enhanced urinary control and improved subjective symptom experiences among patients with PPI.

PPI etiology remains incompletely understood, with risk factors including age, BMI, prostate size, oncologic and surgical factors, and shorter membranous urethral length [20-23]. Despite refined surgical techniques and preoperative precautions, PPI persists, severely impacting QoL, finances, and psychology. Conservative treatment—lifestyle modifications, bladder diaries, and PFMT—is first-line for mild PPI, yet a significant implementation gap exists due to poor adherence influenced by fatigue, transportation, time constraints [24-27], inadequate PFMT technique comprehension, and lower cognitive ability [28]. Supervised rehabilitation outperforms unsupervised approaches [29,30].

Traditional in-person care faces geographical, resource, and adherence limitations. Digital health interventions extend support beyond clinics [31]. This study developed CBISs, which collects multidimensional data (diet, urination, activities, adherence, and perceived severity) to generate individualized RL-driven recommendations through XGBoost with rule-engine integration, continuously optimizing through cross-validation while preventing overfitting.

Several digital therapeutic approaches for PPI have been reported, including cognitive-behavioral therapy–based telehealth interventions for urinary incontinence control and QoL [31,32], mobile health apps combining bladder diary logging with PFMT prompts [33], perioperative telehealth programs with remote monitoring calls [28], WeChat-based health education and extended care services [34,35], social media platforms for patient education and community support [36], and proactive digital health interventions aimed at symptom reduction [37]. These platforms primarily focus on delivering educational content, standardized exercise instructions, symptom tracking, and periodic feedback through telehealth communication, messaging apps, or social media engagement. While such approaches improve accessibility and patient awareness, their rehabilitation protocols are generally static, relying on rule-based prompts, fixed schedules, or clinician-mediated adjustments. Consequently, they have limited capacity to adapt training intensity, content, or timing in response to individual patient trajectories, fluctuating adherence, or delayed and heterogeneous treatment responses.

In contrast, the CBISs introduces an RL–driven, closed-loop behavioral intervention framework that emphasizes continuous personalization rather than predefined content delivery, consistent with recent applications of RL in noncommunicable disease management and personalized medicine [38,39]. By iteratively integrating multidimensional patient data—including voiding behaviors, fluid intake patterns, and training adherence—the CBISs dynamically adjusts rehabilitation guidance in response to individual progress and engagement over time. This adaptive design is particularly suited to behavioral rehabilitation contexts, where patient responses evolve gradually and vary substantially across individuals, representing a key limitation of conventional static or rule-based digital platforms.

From a usability and scalability perspective, the CBISs is designed to translate complex behavioral information into actionable, individualized recommendations while reducing reliance on continuous clinician involvement. Clinicians primarily function as supervisors supported by automated monitoring and alert mechanisms, which may help mitigate workforce constraints in real-world clinical settings. Although this pilot study was not designed to establish definitive efficacy, the observed favorable trends in urinary symptom–related outcomes suggest that RL-driven adaptability may help address common challenges of existing digital rehabilitation approaches, including declining adherence and variable response patterns.

Collectively, these features distinguish the CBISs from existing digital therapeutic platforms by shifting the focus from content-centered delivery to behavior-centered, data-driven optimization. This paradigm more closely reflects the dynamic nature of rehabilitation processes and provides a plausible pathway for improving long-term engagement and personalization in PPI management.

Several limitations warrant consideration. First, transparency and interpretability remain inherent challenges of machine learning–based systems. Although clinician oversight was incorporated into the CBISs workflow, the “black box” nature of RL algorithms necessitates ongoing monitoring to ensure the clinical appropriateness and reliability of generated recommendations. For example, the model may recommend aggressive voiding interval reductions (±15 min) for patients with irregular adherence decay without clearly explaining feature interactions (eg, fluid intake influencing urgency events). In pilot testing, 3 of 16 (18.8%) cases triggered low-confidence alerts routed to clinician override due to unexplained Q value shifts during nocturia spikes, highlighting the need for explainable AI techniques like Shapley additive explanations analysis in future iterations. CBISs relies on patient-reported bladder diaries and potential wearable integration, introducing variability from self-reporting inaccuracies (eg, underreported leakage) or device nonuse (12% dropout in usability testing). Participation may have induced Hawthorne effects, with improvements potentially attributable to heightened attention from research involvement rather than CBISs alone. Additionally, while pilot (n=16) and modeling (n=150) cohorts shared consistent inclusion and exclusion criteria, all participants were motivated outpatients from routine clinical settings, potentially overrepresenting compliant, tech-savvy patients and limiting generalizability to broader PPI populations with lower motivation or compliance challenges. In addition, the system processes large volumes of sensitive patient-level data, underscoring the importance of robust data security, privacy protection, and governance mechanisms for real-world implementation.

The pilot evaluation used a prospective self-controlled pre-post design, which is appropriate for early-stage investigation of interventions requiring high patient engagement and ethical sensitivity. By using patients as their own controls, this design reduced interindividual variability and improved statistical efficiency under small-sample conditions, supporting preliminary exploration of feasibility and potential effect sizes. The concurrent use of objective measures (pad test) and validated patient-reported outcomes (ICIQ-UI SF) further strengthened result interpretation.

Nevertheless, the absence of a concurrent control group limits causal inference, and the observed changes may be partially attributable to nonspecific factors such as time effects, repeated measurement, or expectancy-related bias. The relatively short follow-up period also precludes assessment of long-term effectiveness, sustainability of behavioral change, and delayed adverse effects.

Future studies incorporating appropriate control groups, blinded outcome assessment, and extended follow-up are warranted to establish the definitive clinical value of this intervention. In addition, future work will explore the integration of validated and delayed clinical outcome signals into the RL framework once sufficient longitudinal and controlled trial data become available.

Future studies with complete longitudinal labeling and independent test cohorts will enable formal reporting of model performance metrics such as AUC, *F*_1_-score, and MAE. The lack of a fully formalized mathematical reward function reflects an intentional design choice in this exploratory phase and will be addressed in future work when sufficient validated outcome data become available. Comparative evaluation against simpler baseline models was beyond the scope of this feasibility-focused study and will be addressed in future controlled investigations.

### Conclusions

This study describes the development and preliminary evaluation of a behavioral therapy–based CBISs designed to support personalized rehabilitation for PPI. By integrating multidimensional patient data—including voiding behaviors, fluid intake patterns, PFMT adherence, and patient-reported outcomes—the CBISs applies an RL framework to deliver adaptive, individualized behavioral guidance beyond static information delivery.

Rather than replacing clinician decision-making, the CBISs functions as a digital extension of guideline-based care, with the potential to enhance continuity, personalization, and accessibility of rehabilitation support outside traditional clinical settings. The findings from this early-stage study suggest feasibility and favorable trends, supporting further investigation of this approach.

Future multicenter randomized controlled trials are warranted to address several key research questions including whether (1) RL-driven personalization improves continence recovery, QoL, and adherence compared with standard or nonpersonalized digital rehabilitation; (2) observed benefits are sustained over longer follow-up periods; (3) the system performs across diverse patient populations, clinical environments, and levels of baseline motivation; and (4) adaptive, closed-loop interventions confer incremental value over conventional rule-based digital platforms. Addressing these questions will be essential for defining the clinical effectiveness, generalizability, and implementation potential of the CBISs.

As DTx continue to evolve, systems such as the CBISs may offer a scalable pathway toward precision rehabilitation, supporting individualized recovery trajectories for patients navigating functional challenges following prostate cancer surgery.

## Abbreviations

AI: artificial intelligence
AUC: area under the receiver operating characteristic curve
CBIS: clinical behavioral intervention-supporting system
DTx: digital therapeutics
ICIQ-UI SF: International Consultation on Incontinence Questionnaire–Short Form
MAE: mean absolute error
PCa: prostate cancer
PFMT: pelvic floor muscle training
PPI: postprostatectomy incontinence
QoL: quality of life
RL: reinforcement learning
SM3: ShangMi 3
SM4: ShangMi 4
XGBoost: extreme gradient boosting

## Data Availability

The data that support the findings of this study are available from the corresponding authors upon reasonable request.
